# Biocompatibility Study of a Commercial Printed Circuit Board for Biomedical Applications: Lab-on-PCB for Organotypic Retina Cultures

**DOI:** 10.3390/mi12121469

**Published:** 2021-11-29

**Authors:** Jesús David Urbano-Gámez, Lourdes Valdés-Sánchez, Carmen Aracil, Berta de la Cerda, Francisco Perdigones, Álvaro Plaza Reyes, Francisco J. Díaz-Corrales, Isabel Relimpio López, José Manuel Quero

**Affiliations:** 1 Electronic Technology Group, Department of Electronic Engineering, Higher Technical School of Engineering, University of Seville, Avda. de los Descubrimientos sn, 41092 Seville, Spain; jurbano1@us.es (J.D.U.-G.); fperdigones@us.es (F.P.); quero@us.es (J.M.Q.); 2 Department of Regeneration and Cell Therapy, Andalusian Molecular Biology and Regenerative Medicine Centre (CABIMER), Avda. Américo Vespucio 24, 41092 Seville, Spain; lourdes.valdes@cabimer.es (L.V.-S.); alvaro.plaza@cabimer.es (Á.P.R.); francisco.diaz@cabimer.es (F.J.D.-C.); 3 RETICS Oftared, Carlos III Institute of Health (Spain), Ministry of Health RD16/0008/0010, University Hospital Virgen Macarena, Avda. Dr. Fedriani, 3, 41009 Seville, Spain; irelimpio@gmail.com

**Keywords:** Lab-on-PCB, printed circuit board, organotypic culture, biomedical applications

## Abstract

Printed circuit board (PCB) technology is well known, reliable, and low-cost, and its application to biomedicine, which implies the integration of microfluidics and electronics, has led to Lab-on-PCB. However, the biocompatibility of the involved materials has to be examined if they are in contact with biological elements. In this paper, the solder mask (PSR-2000 CD02G/CA-25 CD01, Taiyo Ink (Suzhou) Co., Ltd., Suzhou, China) of a commercial PCB has been studied for retinal cultures. For this purpose, retinal explants have been cultured over this substrate, both on open and closed systems, with successful results. Cell viability data shows that the solder mask has no cytotoxic effect on the culture allowing the application of PCB as the substrate of customized microelectrode arrays (MEAs). Finally, a comparative study of the biocompatibility of the 3D printer Uniz zSG amber resin has also been carried out.

## 1. Introduction

Although printed circuit board (PCB) substrates are typically used for electronics, the development of devices based on PCB substrates has been the subject of increasing research over the years [1,2]. The reason for this lies in the advantages that PCB implies for biomedical applications and marketability [3,4], such as low cost, commercial availability, and the possibility of integrating electronic circuits with ease. It is important to note that this kind of device combined with microfluidics can perform different laboratory tasks, such as reactions or mixing in a small laboratory with the dimensions of a credit card [4]. Moreover, the possibility to integrate heaters and temperature sensors can provide a chamber with the optimal biological conditions. These devices are named PCB-based lab-on-a-chip or Lab-on-PCB (LoP) devices. Up to now, these devices have been applied to several biomedical fields, such as detection of cell viability [5], molecular diagnosis [6], DNA amplification [7,8], and electrolytes detection [9].

Focusing on one of the most important biomedical applications, electrophysiology, where MEAs are frequently used, different approaches have been developed regarding the design of these devices. Electrostimulation and acquisition of signals from the biological material is accomplished thanks to these MEAs, which have to be just below the cells or organotypic cultures. The insulation material is an important component of the MEAs, since it is in direct contact with the culture. The role of this material is to avoid the electrical contact between cells/tissues and the metal tracks, releasing only the electrodes. A typical insulation material is silicon nitride (SiN), used in many MEAs, for instance, in the standard MEA of multichannel systems [10,11], in the high-density version [12], in the 3D version [13], and in the PEDOT-CNT MEA [14]. In the perforated versions of those MEAs, the insulation material is polyimide [15].

The PCB technology can be a good choice for performing these devices, in both rigid or flexible substrates, although the biocompatibility has to be considered. There are several examples of MEAS manufactured with this technology: a flexible one, using polyimide as both substrate and insulation material [16], and a fabricated one, using PCB (Eco MEA) with Elpemer®2467 or PSR-4000 GP01EU (Peters, Kempen, Germany and Taiyo America, Carson City, NV, USA, respectively) [17] as insulation material. Other designs can be found in state of the art such as the PCB-based 3D MEA described on [18]. This MEA has gold electrodes covered with SU-8 as an insulation layer. A different version of PCB-based MEA is reported on [19], where 3D gold microelectrodes are embedded on polydimethylsiloxane (PDMS). The biocompatibility of this MEA was demonstrated for retinal explant from a retinitis pigmentosa mouse-model. However, in the latter examples the fabrication process is quite complex.

Our goal is the fabrication of a MEA on PCB for specific biomedical applications, which is the evaluation of neuroprotective strategies for retinal degenerative diseases, and we expect to achieve an easy, reliable, and low-cost design that can be adjusted to a wide range of applications. The objective is to give the researchers the possibility to develop their customized MEAs, at low cost, instead of being limited to the current commercial MEAs or investing a higher cost for suggesting a design. Regarding the materials, the rigid PCB substrate is composed of copper and FR4, where the copper is typically covered with gold. Standard and commercial PCB technology includes a solder mask as an insulation layer. The specific insulation layer depends on the PCB manufacturing company. Polyimide is a biocompatible material used as a flexible PCB insulation layer, particularly for retinal implants [20]. In addition, a typical solder mask of flexible substrates (PSR-9000 FLX03G LDI, Taiyo America, Carson City, NV, USA) seems to be biocompatible [21]. However, we plan to use commercial rigid PCB substrates for manufacturing MEAs, from a specific company [22]. This company uses specifically a white solder mask (PSR-2000 CD02G/CA-25 CD01, Taiyo Ink (Suzhou) Co., Ltd., Suzhou, China) [22], which has to be tested for biocompatibility, which is the main aim of this work.

Regarding biocompatibility, it is convenient to perform the study for the specific application that will be implemented. In our case, the MEA will be applied to the study of retinal degenerative diseases. Retinal degenerative diseases range from classic genetic diseases such as retinitis pigmentosa to complex traits that constitute most of the blinding diseases in the industrialized world such as macular degeneration, glaucoma, and diabetic retinopathy. In every case, neurodegeneration and cell death ultimately produce low vision or blindness. The high impact of sight loss at the individual and societal level generates a demand for knowledge on the disease pathways and the development of new therapeutic approaches. Researchers look for neuroprotection to fight cell death and regenerative medicine methods to improve vision.

Organotypic culture has been applied to the study of retinal biology, including postnatal retinal development, factors influencing the retinal degenerative process, and the assay of candidate therapeutic molecules that may modify the degenerative process. The current retinal organotypic culture methodology was described for neonatal [23] mice retinas and adapted to adult mice retinas by Müller et al. [24]. The utility of the organotypic methodology benefits from the similarity of the retinal explant to the natural tissue compared to the cell culture of isolated retinal components while allowing the researchers to keep a tightly controlled experimental condition that sometimes is not possible in in vivo animal experiments. Additionally, an organotypic culture allows for continuous monitoring of the tissue via integrated optics and/or MEAs. Previously, some studies achieved electrical stimulation and recording applied to therapeutic evaluation, viability assessment, and functional characterization of retinal explants and organoids [19,25,26,27,28,29] with the use of MEAs as a fundamental tool.

It is also important, for the adequate design of the biocompatibility test, to consider the microfluidic platform where the organotypic culture will be located. In our case, the MEA will be integrated inside a lab-on-chip (LoC), so the biocompatibility tests have to be designed taking into account the organotypic culture that will be located inside a closed system. The standard retinal organotypic culture is based on a permeable Millicell membrane as support of the tissue, in which the retinas stay in a liquid–air interface. In our study, we have first compared the performance of two different open/static culture systems, with the Millicell membrane as a control, to evaluate the biocompatibility of the materials. In a second approach, we have tested the viability of the retinal tissue in the same two systems but in closed configuration, which is more similar to our goal of a stand-alone LoC system.

In this paper, a Lab-on-PCB for long-term organotypic retinal explant culture has been developed and used to demonstrate the biocompatibility of commercial rigid PCB substrates, specifically for white solder masks of the company JLCPCB (PSR-2000 CD02G/CA-25 CD01, Taiyo Ink (Suzhou) Co., Ltd., Suzhou, China) [22]. The ability to sustain the biological material in culture onto the commercial PCB implies the possibility of customization of MEAs with different dimensions, the number of electrodes and different shapes, and the use of an open-source and user-friendly software, just to be ordered to a fabrication company.

## 2. Culture Chambers

In order to study the biocompatibility of the solder mask, a PCB culture chamber, completely covered with solder mask, and with a frame of PMMA, has been manufactured. For comparative purposes, a different chamber has been also designed. In this case, it is based on a commercial glass microelectrode array (MEA), plus a frame of 3D printer resin. These culture platforms are described below.

### 2.1. PMMA/PCB Culture Chamber

This platform was designed using Klayout open-source software. The chamber dimensions, adapted to the MEA, correspond to 32 × 32 mm.

**Figure 1:**
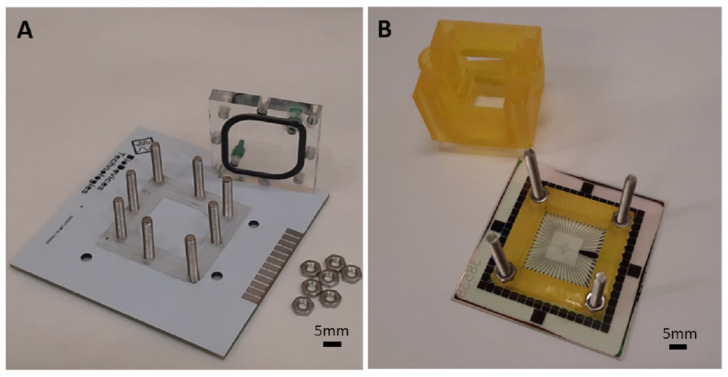
Culture chambers. (**A**) Culture chamber composed of PCB as substrate and a fluidic system made of PMMA. (**B**) Culture chamber composed of MEA from MultiChannel systems (MCS) as substrate and a fluidic system made of Uniz zSG amber resin.

The culture chamber was composed of a cover and a base, both of them made of PMMA over a PCB substrate. This substrate was completely covered with an insulation layer, a solder mask, that was be in direct contact with the biological material, so it was mandatory to study its biocompatibility. The PCB substrate was a sheet with a height of 1.6 mm, which was covered, entirely, with a white ink solder mask (PSR-2000 CD02G/CA-25 CD01, Taiyo Ink (Suzhou) Co., Ltd., China) [22] used as an insulation layer. These parts can be seen in Figure 1A.

The base of this platform was designed as an eye-shaped inner hole with a diameter of 20 mm and eight additional holes with 3 mm diameter, distributed around the external frame, for screws. These screws were used to ensure the correct alignment and coupling of both the cover and the base.

The cover includes two holes with a diameter of 2.7 mm to be used as inlet and outlet ports. Male mini-luer fluid connectors were inserted in these holes of the cover. In this part of the platform, an eye-shaped milled channel was also included where a sealing rubber ring (1.6 mm diameter) was attached to ensure correct isolation of the inner chamber and to avoid any leakage of liquid. In addition, eight holes of 3 mm diameter were added at the same position as the ones in the base, in order to align both parts of the platform with screws.

Regarding the fabrication process, an automated CO_2_ laser milling machine was used for fabricating both the cover and the base, and 2 mm-thick and 5 mm-thick PMMA sheets were used to build the cover and the base, respectively. Once the screws were coupled to the base, the PCB substrate was attached using a biocompatible epoxy glue, EPO-TEK®301 (Epoxy Technology, Billerica, MA, USA). This adhesive was also used for the attachment of the sealing ring to the eye-shape milled channel of the cover.

### 2.2. 3D Printer Resin/Commercial Glass MEA Culture Chamber

The design and manufacturing of this fluidic platform using a 3D printer resin have been previously published [30]. Briefly, a 3D model for organotypic cultures composed of two parts was designed using Fusion 360 software from Autodesk. The base and the fluidic platform were coupled with screws, for its integration to a commercial MEA. These parts can be seen in Figure 1B. The fluidic platform has two integrated connectors: inlet and outlet ports. These ports communicate with the culture chamber at different heights, that is, the output port is placed higher than the inlet port. This configuration creates a tilted top surface with an angle of 10º between both openings that helps the removal of air bubbles. The culture area was defined to be 21 × 21 mm, and the external dimensions of the base were 32 × 32 mm.

Regarding the manufacturing process, the Anycubic Photon 3D printer was used with the Uniz zSG Amber resin. This material will be in direct contact with the culture media, where the retinas are located, so its biocompatibility has also to be studied, but it will not be in direct contact with the retinas, so the biocompatibility test should replicate this specific situation. The isolation of the culture chamber was ensured by attaching a glass coverslip (tilted surface) in the window on the fluidic structure and using an ethylene-vinyl acetate (EVA) film, between the base and the fluidic platform. The adhesion of the base to the MEA surface and the attachment of the glass coverslip to the fluidic platform were performed using EPO-TEK®301 (Epoxy Technology, Billerica, MA, USA).

The substrate used for this culture platform was the commercial MEA 60MEA500/30iR-Ti (MultiChannel Systems MCS GmbH, Reutlingen, Germany). This model features 60 titanium microelectrodes that are 30 μm diameter and an interelectrode distance of 500 μm, distributed in a 6 × 10 array. Finally, the insulation layer of this substrate is silicon nitride.

## 3. Materials and Methods

For the analysis of the biocompatibility of the materials tested, mouse retinal explants were incubated within both chambers for 48 h. Different tests have been carried out to analyze the biocompatibility using tissue from young and adult mice. Animal care and experimentation followed the Association for Research in Vision and Ophthalmology Statement for the Use of Animals in Ophthalmic and Vision Research and the guidelines of the European Union Directive 2010/63/EU on the protection of animals used for scientific purposes and was approved by Institutional Animal Ethics Committee.

Regarding the retinal explants, the study focuses on the neuroretina, a multilayered tissue composed of different types of neurons and glial cells. The photoreceptors, which are the photosensitive neurons, constitute the outmost layer, and the ganglion cells that transmit the information to the brain are in the innermost layer. Retinal explants were prepared from C57BL/6 wild-type mice of ages ranging from 14 days post-natal to 60 days (adult). A standard aseptic dissection technique [31] was used, in which retinal pigment epithelium was peeled off from the neuroretina, and each of the neuroretinas was flattened from their original spherical shape by four scissor cuts, rendering a “flower” shape known as flat mount. To avoid cellular damage induced by excessive manipulation and to ease the placement of the tissue in the system, the dissected neuroretinas were mounted onto a perforated polyvinylidene fluoride (PVDF) frame. Neuroretinas were arranged in each of the systems with the photoreceptor layer facing toward the material being tested for biocompatibility, with the ganglion cell layer facing upward. Interphase with the culture media was kept through a permeable Millicell (Millipore PIOCM0RG50) membrane on top pf the PVDF/retina structure. The biological material was fed with culture media (Neurobasal-A (Gibco 10888022), 2% B27 (Gibco 7504044), 1% N2 (Gibco 17502048), 1% pen/strp (Gibco 15140122), and 0.4% Glutamax (Gibco 35050038)).

**Figure 2:**
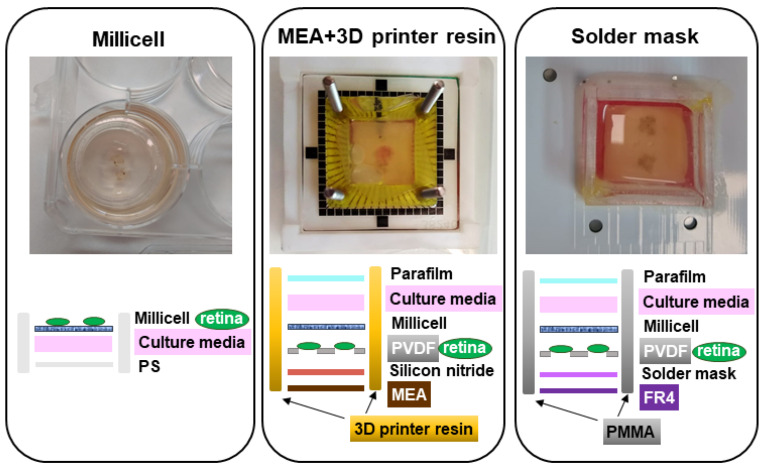
Images of the three different open culture systems and schematic representation of the setting for each case.

In a first approach to measure the cell viability of retinas cultured onto the tested materials, a static/open system was set using 14-days postnatal retinas. In this case, taking into account the fact that the system is a static/open mount, the more adequate control is to maintain mouse retinas onto a standard Millicell cell culture insert inside a regular cell culture plate. However, this is not a proper control for a closed/continuous flow system, since the cultures’ conditions are too different to be compared. For comparative purposes for the test in closed/continuous flow, we have used the commercial MEA of MultiChannel. A microfluidic platform is manufactured in a 3D printer and glued to the MEA. For the biocompatibility test of this setting, the only material to be studied is the 3D resin, since the rest of materials (Silicon Nitride, PVDF, and Millicell) are known to be biocompatible. Moreover, the resin is only in contact with the culture media and not with the tissue, so the biocompatibility experiment has to replicate this situation, and in this case it will not be relevant to test the biocompatibility by culturing the retinas in direct contact with the material. The three different open culture systems can be seen in Figure 2.

Explants were laid onto the PCB, completely covered with solder mask, (with a culture chamber made of PMMA), and laid into a resin-based culture chamber (attached to a biocompatible MEA as culture substrate), covered by 1 mL of culture media and maintained into a standard cell culture incubator at 5% CO_2_, 20% O_2_ atmosphere and 37 °C for 48 h. On top, a sterile sheet of Parafilm was used to avoid evaporation of the culture media. A standard Millicell culture insert was used as a control for cellular viability of the retinal explants. Once the culture time was finished, each retina was carefully retrieved from its culture system. In this experiment, dead retinal cells were stained using ethidium homodimer-1 and counterstained with calcein (live/dead viability/cytotoxicity kit for mammalian cells, molecular probes MP 03224) by incubating the tissue in 0.4% ethidium homodimer, 0.125% calcein in D-PBS (Merk 59331CC) for 30 min at 37 °C before proceeding to tissue disaggregation to obtain single cells. Retinas were incubated for 5 min with 100 μL of a mixture of proteolytic and collagenolytic enzymes (Accutase, Stemcell 07920) at 37 °C, followed by soft agitation using 10 up-down pipet cycles with a 1 mL micropipette, and 100 μL of a 1 mg/mL DNase I (Roche 10104159001) solution in D-PBS (Merk 59331C) was added and incubated for additional 5 min at 37 °C. Soft agitation was performed again five times, and the cell suspension was filtered through a 40 μL cell strainer (Falcon 352340). Cytometry buffer (2% FBS (ThermoFisher Scientific 16140071), 1 mM EDTA (Sigma E5134) in D-PBS) was added to complete 1 mL. Biocompatibility was tested via flow cytometry using a FACSCalibur flow cytometer (BD Biosciences, San Jose, CA, USA) equipped with 488 nm and 635 nm lasers. Analysis of the data was carried out using FlowJo v10.7 software (BD Biosciences, San Jose, CA, USA) and will be discussed in Section 4.

**Figure 3:**
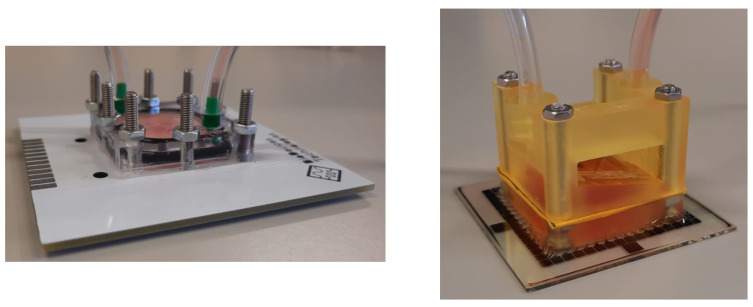
The closed systems evaluated are shown: on the right side, the commercial MEA + 3D printer resin, and on the left, the PCB system.

In order to evaluate biocompatibility of PCB and resin separately, as stand-alone culture systems independent from a cell incubator, a closed system was set for the PCB-PMMA LoP and for the MEA-resin lab on chip (LoC) systems (shown in Figure 3). A continuous flow of cell culture media was sustained by a syringe pump (new era pump systems NE-1000) at a 3.6 μL/min rate and maintained at a 37 °C temperature in a temperature-controlled chamber. Culture media was equilibrated in 5% CO_2_, 20% O_2_ atmosphere before starting explant feeding. Adult mouse retinas and a different staining technique were used in this occasion. Tissue was disaggregated before staining, and dead cells were labeled with 2-(4-amidinophenyl)-1H-indole-6-carboxamidine (DAPI; 1:2500 in cytometry buffer) and measured by flow cytometry. DAPI is a fluorescent molecule that binds strongly to DNA but cannot pass through an intact cell membrane and, therefore, preferentially stains dead cells.

Taking into account that our main focus is the preparation of LoC systems aimed to study retinal degeneration and to test possible means of neuroprotection, we are specifically interested in the retinal cells that degenerate in our mouse model, which are the rod photoreceptor cells. Rod cells express rhodopsin. To determine the preservation of the rod cells after explant culture in these systems, we used cells from the closed system experimental conditions and labeled them with an antibody against rhodopsin.

For the flow cytometry analysis of rhodopsin-expressing cells, single cells were incubated for 30 min with Violet Live/Dead fixable stain (ThermoFisher Scientific, Waltham, MA, USA) and fixed with BD Cytofix/Cytoperm^TM^ kit (BD Biosciences, San Jose, CA, USA). After fixation and permeabilization, cells were stained with a primary antibody against rhodopsin (Abcam # ab190307, 1:100) for 30 min on ice, followed by washes and staining with donkey anti-mouse Alexa Fluor 488 (ThermoFisher #A-21202, 1:1000) secondary antibody for another 30 min on ice. Fluorescence minus one (FMO) controls were included for each condition to identify gate-negative and -positive cells. Stained cells were analyzed using a LSRFortessa X-20 flow cytometer equipped with 488 nm, 561 nm, 405 nm, and 640 nm lasers (BD Biosciences, San Jose, CA, USA). Analysis of the data was carried out using FlowJo v10.7 software (BD Biosciences, San Jose, CA, USA). Only single cells data was considered for the analysis, excluding cell fragments and cell aggregates.

## 4. Experimental Results

**Figure 4:**
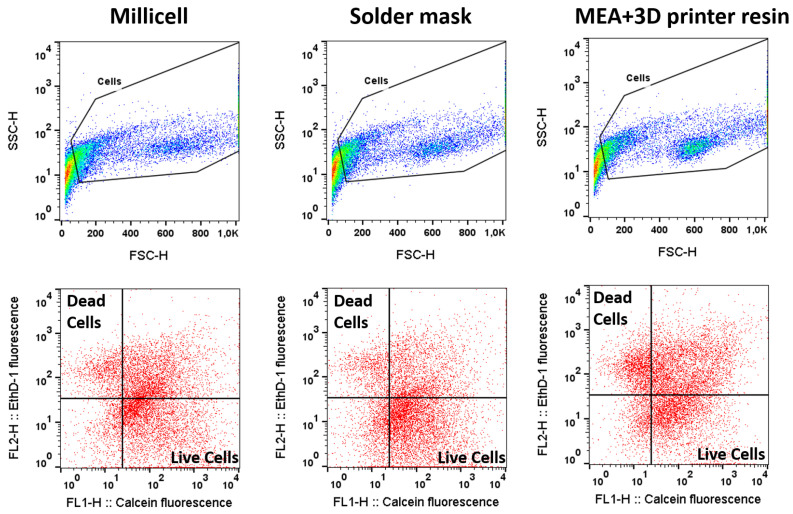
Biocompatibility measured in open culture systems. Representative images of cytometer acquisition data of cells cultured on Millicell insert, on solder mask, and on MEA + 3D printer resin, as shown from left to right. The upper row shows the gating of single cells. The lower row shows calcein fluorescence in the x-axis and ethidium fluorescence in the y-axis, corresponding to live and dead cells, respectively.

**Figure 5:**
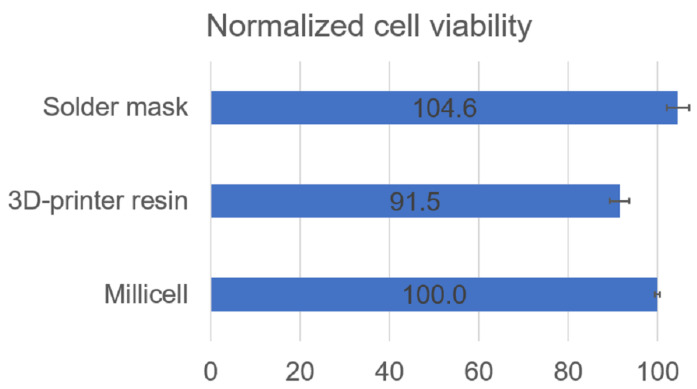
Biocompatibility measured in open culture systems. Bar plot displaying the viability of retinal cells after being cultured with three different materials for 48 h in open systems. Percentage of viable cells after culture of retinal explants onto Millicell membrane, PCB, and MEA + 3D printer resin. The error bars represent the standard deviation.

For the analysis of the biocompatibility of the solder mask with retinal explants, mouse retinas were prepared with a standard explant technique and cultured for two days in contact with this material. As previously commented, we first used an open configuration of the culture system, and organotypic culture was performed into a standard cell culture incubator. A regular Millicell retinal explant culture was used as control, in the first test, to compare cell viability. Additionally, a MEA + 3D printer resin was also studied, for comparative purposes in the following experiments in closed configuration. Data of Figure 4 and Figure 5 correspond to 10,000 recorded cells from each of two retinas in each condition.

Flow cytometry analysis of the retinal cells stained with Ethidium homodimer-1 and calcein A shows that the solder mask preserves retinal cell viability in a similar proportion to the standard Millicell insert used as a control. We found that retinal explants cultured in the MEA + 3D printer resin system present a slightly lower cell preservation of cell viability. However, we considered that this difference in cell viability was small enough to still consider the MEA + 3D printer resin system a potentially good experimental setting for subsequent testing. Considering our aim and future experimental purposes, both PCB-solder mask and MEA + 3D-printer resin sustain the culture of retinal explants adequately to test neuroprotective agents and to study the degenerative process in these culture conditions. Altogether, these results indicate that neither the solder mask nor the resin exert a cytotoxic effect on cell viability, and that both materials displayed acceptable levels of biocompatibility.

**Figure 6:**
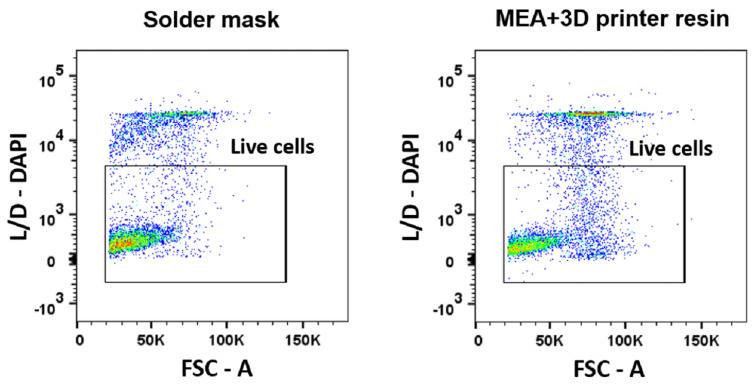
Biocompatibility measured in closed culture systems. Representative dot plots of cytometer acquisition data of cells cultured in PCB in a closed system and resin-based LoC system. Dead cells are positively stained for DAPI, which is displayed in the Y-axis.

**Figure 7:**
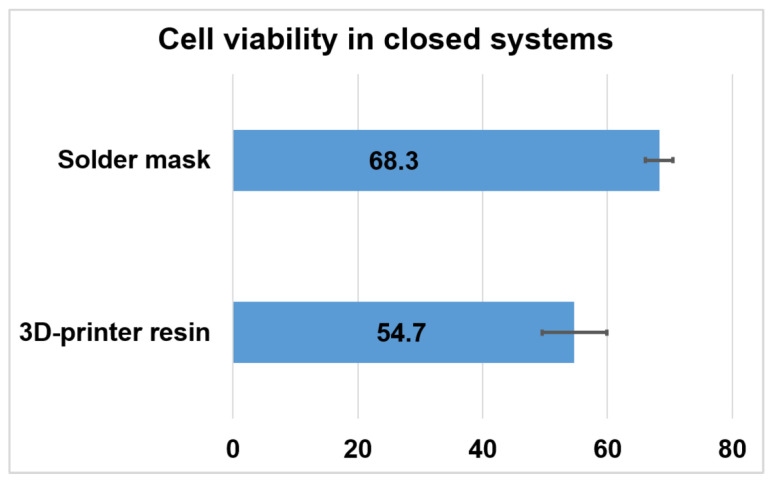
Biocompatibility measured in closed culture systems. Bar plot displaying the viability of retinal cells after being cultured with two different materials for 48 h in closed systems. Percentage of viable cells after culture of retinal explants in autonomous systems onto PCB, and onto a resin-based LoC. The error bars represent the standard deviation.

To replicate this test in an autonomous LoC setting, the retinal explants were cultured for 48 h in a closed configuration outside a cell culture incubator (Figure 3), and the biological material was fed via continuous pumping of culture media. Then, we used flow cytometry to compare the viability of retinas cultured in the PCB-solder mask system (in direct contact with the solder mask) to the viability of retinas cultured on a closed system composed by a commercial MEA and a microfluidic platform made of 3D printer resin (where retinas are in direct contact with the silicon nitride layer of the MEA). Data of Figure 6 and Figure 7 correspond to 10,000 recorded cells from each of three retinas for each condition.

Preservation of the biological material was shown even using adult retinas, which usually present a lower viability in explant culture. In concordance with the results of the previous experimental setting, cell viability preservation was found to be slightly higher when retinal explants were cultured in contact with the solder mask compared to the MEA-3D-printer resin set up.

The long-term goal of this study is the development of a functional LoC system for testing the effect of neuroprotective agents on photoreceptor degeneration occurring in model systems of retinitis pigmentosa, such as the rd10 mice. Therefore, we specifically tested the viability of rod cells in the aforementioned closed culture systems. The specific cell population affected in the rd10 mice model is rod photoreceptor cells, which express rhodopsin. To determine the preservation of the rod cells after organotypic culture into this system, we quantified the number of rhodopsin-positive cells after fixing and staining them with a rhodopsin antibody.

**Figure 8:**
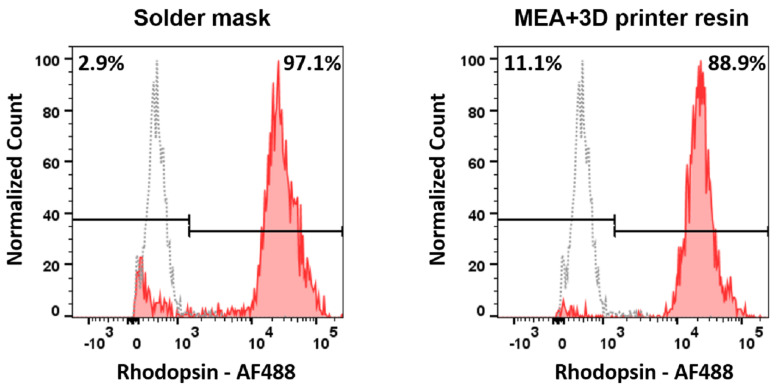
Histograms displaying the percentage of rhodopsin positive cells after culturing retinal explants onto PCB system vs. commercial MEA and 3D printer resin. Fluorescence minus one control (FMO) is displayed in gray as a negative control used for setting the quantification gates.

Our results (Figure 8) show good preservation of rod cells that is similar in both conditions tested, with 97.1% of rhodopsin positive events for the cells cultured onto PCB and 88.9% for the resin-based device. Therefore, our data indicate that both the PCB and the resin have no specific toxicity for the rod photoreceptor cells, which were in contact with these materials for 48 h. In line with our previous results, PCB-solder mask presents a better preservation of retinal cells—in this case, specifically rod photoreceptors—than 3D printer resin. However, the yet good rod photoreceptor conservation observed in the MEA-3D printer resin set up (>88%) demonstrates that this system can still be considered a good candidate for organotypic retinal culture.

## 5. Conclusions

Different experiments of cell viability have been performed to evaluate the biocompatibility of the solder mask of the PCB. Moreover, the biocompatibility of a printable resin has also been carried out. Both materials were in contact with the retinal explant culture, and the results show both of them are compatible with retinal cultures.

Our data support the use of PCB (covered with this solder mask) as a component of organotypic culture systems and, more specifically, in systems designed to evaluate retinal explants for the study of photoreceptor degeneration and neuroprotection. In Lab-on-PCB, where the integration of microfluidics and electronics is required, this is an essential point. Therefore, these results sustain the future target of fabricating the MEA on PCB, for studies on regenerative medicine to develop methods to improve vision. However, if the dimension requirements about electrodes and tracks for a specific application are more demanding than the commercial PCB manufacturer offers, the MEA of MultiChannel Systems, integrated to a microfluidic platform made of 3D printer resin, could serve as an alternative, although at a higher economic cost. Another significant advantage is that it could be considered an open-source design Klayout and FreeCad software are used for the design. A stand-alone Lab-on-PCB system in which the retinal explants can be constantly monitored for the effect of different stressors or neuroprotective agents/conditions would provide valuable information on the neurodegenerative process that leads to cell death and sight loss. It would also provide a tool to test different approaches against cell death caused by retinal degenerative diseases. Specifically, we plan to use these devices to test the effect of different doses of electrostimulation, the different methods of gene therapy to address degenerating cells in the retina, and new agents with neuroprotective properties that will be added to the circulating culture media. Once the utility of these systems is established using retinal explants, we plan to apply this technology to the evaluation of retinal organoids derived by in vitro differentiation from cells of human healthy donors and patients, avoiding the use of laboratory animals and obtaining information closer to the real human patient. The use of the Lab-on-PCB would not be restricted to research in the retinal field; it could be expanded to wider biomedical applications, culturing different cell types, other organotypic cultures, or patient-derived organoids.

The possibility of developing low-cost MEAs, customized to other biomedical applications, with a cost about 10 times cheaper than commercial MEAs with similar characteristics, will offer the researchers great flexibility for designing their own MEAs and experimental settings.

## Data Availability

Data is contained within the article.

## Abbreviations

The following abbreviations are used in this manuscript:

PCB	Printed Circuit Board
MEA	Microelectrode array
LoP	Lab-on-PCB
PMMA	Polymethylmethacrylate
EVA	Ethylene-vinyl acetate
LoC	Lab-on-chip
PVDF	Polyvinylidene fluoride
PS	Polystyrene
FMO	Fluorescence minus one
